# MyastheniaGravis(MG) in a patient with Juvenile Idiopathic Arthritis

**DOI:** 10.1186/1546-0096-9-S1-P185

**Published:** 2011-09-14

**Authors:** Christiaan Scott, Asgar Kalla

**Affiliations:** 1 Red Cross War Memorial Childrens Hospital, Cape Town, South Africa; 2 Department of Rheumatology, University of Cape Town, Cape Town, South Africa

## Introduction

Myasthenia Gravis associated with Juvenile Idiopathic Arthritis has been reported in 5children with various subtypes of JIA [1,2].

## Methods

We present a 17year old girl known with Rheumatoid Factor Positive Polyarticular Juvenile Idiopathic Arthritis for 4 years who developed Myasthenia Gravis while on therapy with Methotrexate, Prednisone and Ibuprofen.

## Results

This patient presented to the emergency room with a respiratory infection. She had been feeling weak and had noticed tongue weakness and difficulty swallowing, which had worsened significantly since the respiratory infection.

On examination she was found to have clinical signs of Right Middle Lobe pneumonia and was found to be weak, especially in her proximal muscle groups. She had bilateral ptosis as well as facial weakness. She had active arthritis in multiple joints. Despite intravenous antibiotics and full supportive management she deteriorated rapidly, and within 12 hours required intubation and ventilation.

The patient was found to have high ACH receptor antibodies and responded dramatically to pyridostygmine therapy, confirming the diagnosis of MG. High prednisone and azathioprine have been added to her regime.

## Discussion

Myasthenia Gravis is a rare association with JIA. The majority of cases appear to be associated with oligo-articular JIA. This patient presented after an acute infection and a recent worsening in her JIA symptoms.
